# Symptomatic Medial Bone Excrescence in the Distal Phalanx of the Hallux after the First Metatarsophalangeal Joint Arthrodesis: A Case Report and Radiographic Reviews

**DOI:** 10.1155/2021/6035784

**Published:** 2021-11-17

**Authors:** Yuji Maenohara, Ryutaro Takeda, Song Ho Chang, Yasunori Omata, Sakae Tanaka, Takumi Matsumoto

**Affiliations:** Department of Orthopaedic Surgery, Faculty of Medicine, The University of Tokyo, 7-3-1 Hongo, Bunkyo-ku, Tokyo 113-8655, Japan

## Abstract

Medial bone excrescence at the base of the distal phalanx of the hallux is a common manifestation which is rarely painful. In this case report, we described the first case of the excrescence becoming symptomatic one year after a metatarsophalangeal (MTP) joint arthrodesis of the great toe in a 74-year-old female. The medial bony excrescence which was obscure preoperatively became obvious postoperatively in the anteroposterior foot radiographs. The patient was successfully treated by an excision of the excrescence. In order to clarify the pathology of the excrescence, we reviewed the radiographs with respect to the excrescence before and after hallux surgeries including 97 metatarsal osteotomies and 33 MTP joint arthrodesis. The width of the excrescence measured in the anteroposterior foot radiographs displayed substantial increment one month after the hallux surgeries (osteotomy group: 0.9 ± 0.7 vs. 1.5 ± 0.7 mm, *p* < 0.01; arthrodesis group: 1.3 ± 0.8 vs. 1.8 ± 1.0 mm, *p* < 0.01). However, there was no significant difference in the width of the excrescence between one month after surgery and at the most recent follow-up of around 20 months in average after the surgery (osteotomy group: 1.5 ± 0.7 vs. 1.4 ± 0.7 mm, *p* = 0.62; arthrodesis group: 1.8 ± 1.0 vs. 1.8 ± 0.7 mm, *p* = 0.37). The present case and our radiographic review suggested that the postoperative medial bony excrescence was not the result of change of position of the preexisting excrescence. The correction of pronation deformity through hallux surgeries could emphasize the medial bony excrescence and cause symptomatic irritation upon shoe contact.

## 1. Introduction

**Figure 1:**
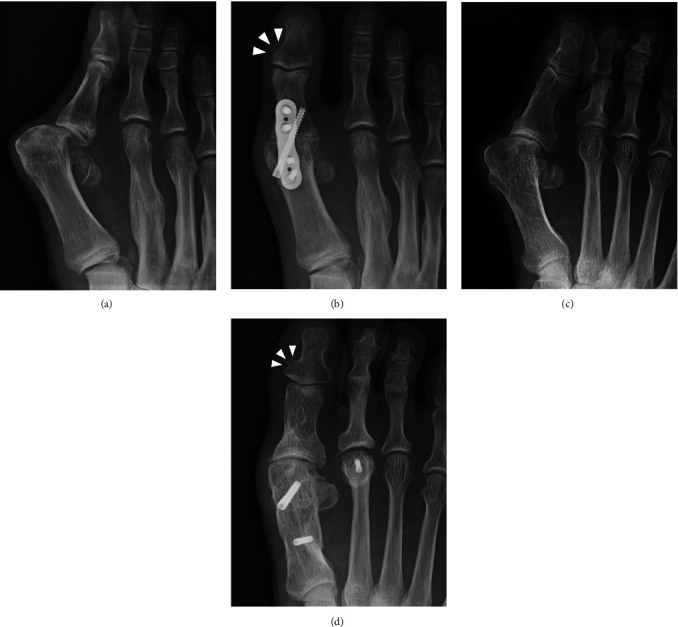
Representative anteroposterior foot radiographs of the cases showing increased width of medial bone excrescence at the distal phalanx of the hallux after the metatarsophalangeal arthrodesis (a, b) or metatarsal osteotomy (c, d) performed to rectify hallux valgus deformity. Arrowheads indicate the protruded bone excrescence after the procedures.

The medial bone excrescence at the base of the distal phalanx of the hallux does not often garner attention as it is rarely symptomatic. Histologically, it is considered to be reactive bone formation and is present in most people with a prevalence rate of up to 88.5% [1]. However, there is a case report in which excrescence became symptomatic after corrective osteotomy for hallux valgus deformity was performed [2]. We present a case in which the excrescence became symptomatic after hallux metatarsophalangeal (MTP) joint arthrodesis was conducted. While speculating on the probable reasons for the development of the new symptoms due to the excrescence after hallux procedures, we noticed that the excrescence that was earlier obscure in the preoperative foot radiograph became conspicuous after surgery in some patients (Figure 1). Because the hallux valgus is a multiplanar deformity including the pronation deformity of the first ray [3], the surgical correction of the deformity might alter the position of the excrescence. However, there has been no literature to explain whether the medial bony excrescence is caused by postoperative growth or rotational change of position of the preexisting excrescence. Therefore, we reviewed the series of foot radiographs before and after hallux surgeries to resolve this question.

## 2. Case Report

**Figure 2:**
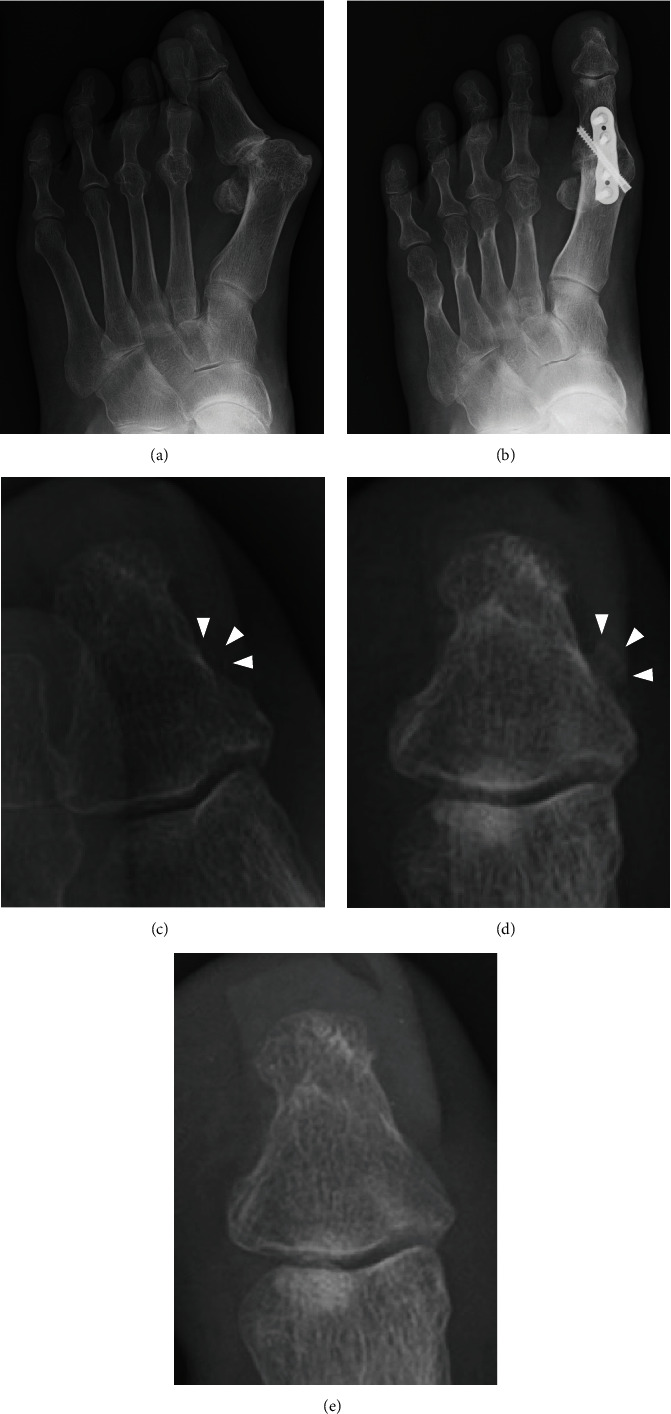
A case of symptomatic medial bone excrescence of the distal phalanx of the hallux after the metatarsophalangeal joint arthrodesis. Anteroposterior foot radiographs before surgery (a) and one year after surgery (b). Magnified images showing obscure excrescence before surgery (c), defined excrescence after arthrodesis (d), and disappeared excrescence after excision (e). Arrowheads indicate the bone excrescence.

The patient was a 74-year-old female with a 32-year history of seropositive rheumatoid arthritis (RA). The patient underwent the first MTP joint arthrodesis for hallux valgus deformity and MTP joint destruction due to RA. The MTP joint arthrodesis was performed through dorsal longitudinal incision using flat cut joint preparation technique [4]. The arthrodesis site was fixed with a headless compression screw and a dorsal locking plate. Hallux valgus angle and intermetatarsal angle between 1st and 2nd improved from 48 to 16 degrees and from 21 to 16 degrees after the surgery, respectively (Figure 2). Although the postoperative course was uneventful, the patient began to complain of localized pain at the medial aspect of the distal phalanx of the hallux during walking with shoes, one year after the procedure. Weight-bearing anteroposterior foot radiographs revealed increased width of medial excrescence at the base of the distal phalanx of the hallux as compared to the preoperative radiographs. Since there was tenderness on palpation and callosity just above the excrescence, the symptom was considered to be caused by this excrescence. Surgical excision of the excrescence was performed with the removal of the metal implant for the MTP joint arthrodesis (Figure 2). Thereafter, the pain disappeared, and there was no recurrence of the symptom at the recent follow-up that was conducted two years after the excision.

## 3. Discussion

A bony excrescence at the medial aspect of the base of the distal phalanx of the hallux is a frequent radiographic finding; however, on rare occasions, it becomes symptomatic. In a review of 157 foot radiographs by Lee et al., including 117 adult and pediatric patients who visited the hospital with foot problems, a specific bone excrescence was observed in 88.5% of the feet [1]. In this study, an outgrowth of 0.4 mm or less was judged as negative excrescence and that of 0.5 mm or more as positive. Another radiographic study by Montiel et al. analyzed unilateral feet of 254 patients who visited the clinic with foot problems and reported a prevalence of 51.9% excrescence, with no description about the judgment criterion [5]. In the present study including a specified cohort of patients who underwent surgery for their hallux disorders, excrescence was observed in 72% of the patients preoperatively and in 93% cases, one month after the surgery as per the judgement criterion by Lee et al. [1].

The clinical implication of the presence of medial excrescence of the base of the distal phalanx of the hallux has been proposed in a case report by Villas et al. that indicated preexisting bony excrescence becomes symptomatic after bunion surgery [2]. This case report by Villas et al. described a 54-year-old woman who developed a painful compression lesion localized to the excrescence after a scarf and Akin osteotomy, which was aggravated by shoe contact and direct pressure. This was successfully treated by surgical excision of the excrescence [2]. Villas et al. attributed this postoperative complication to overcorrection of the hallux deformity with a change in the hallux valgus angle from 33° to 8° and the intermetatarsal angle from 12° to 2°. However, we did not recognize overcorrection of the first ray in the present case.

The bony excrescence at the medial aspect of the base of the distal phalanx of the hallux has been identified as a reactive bone across various literature, suggesting chronic or repetitive stress as a causative factor [1, 5]. In proof of this theory, the absence of excrescence in the juvenile population has been reported [1, 5]. Lee et al. reported the absence of excrescence in the radiographs from patients younger than 18 years. This study also noted a significant correlation between the width of the excrescence and the age of the patient (*r* = 0.57, *p* < 0.0001) in the cohort consisting of 117 patients (6-81 years, mean 37.5) [1]. Montiel et al. reported in their radiographical study which included the patients aged between 2 and 81 years that the prevalence of the excrescence was 1.3% in the patients under 16 years and 65% in those over 60 years [5].

**Figure 3:**
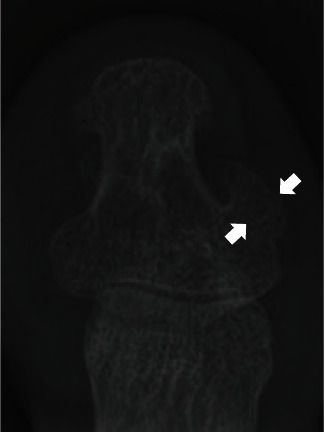
Measurement of the maximal width of the medial bone excrescence of the distal phalanx of the hallux (arrows).

As for the postoperative symptomatic medial bone excrescence, there has been no discussion about its pathology. Thus, we investigated the width of the excrescence in the foot radiographs and compared the results before and after hallux surgeries. Radiographic measurements were performed using the preoperative, one month postsurgery, and most recent anteroposterior weight-bearing foot radiographs for the patients who underwent hallux surgeries including metatarsal osteotomy (osteotomy group, 97 cases in 82 patients) and MTP joint arthrodesis (arthrodesis group, 33 cases in 29 patients) between January 2015 and December 2019 in our facility. The mean follow-up periods were 22.3 ± 13.4 months in the osteotomy group and 20.3 ± 11.2 months in the arthrodesis group. The maximum width of the excrescence in the mediolateral direction was measured as per the method described by Lee et al. (Figure 3) [1]. The patients' backgrounds and radiographic measurements are shown in Table 1. The comparison between the preoperative condition and one month after surgery demonstrated the significant increase of the width of excrescence after surgery in both the groups (osteotomy group: 0.9 ± 0.7 vs. 1.5 ± 0.7 mm, *p* < 0.01; arthrodesis group: 1.3 ± 0.8 vs. 1.8 ± 1.0 mm, *p* < 0.01). There was no significant difference in the width of excrescence between one month after surgery and at the most recent follow-up in both the groups (osteotomy group: 1.5 ± 0.7 vs. 1.4 ± 0.7 mm, *p* = 0.62; arthrodesis group: 1.8 ± 1.0 vs. 1.8 ± 0.7 mm, *p* = 0.37). These radiographic reviews suggested that the postoperative medial bony excrescence was not the result of postoperative growth but just a rotational change of position of the preexisting excrescence following the surgeries. Surgeons must be aware that the excrescence arising from the medial border and plantar aspect of the distal phalanx can be underestimated in the foot with hallux valgus.

To the best of our knowledge, this report is the first to identify a case in which the preexisting asymptomatic excrescence became symptomatic after the MTP joint arthrodesis. There were several possible causes of the preexisting asymptomatic excrescence becoming symptomatic after the procedure: (1) the change in position of the excrescence after the procedure, (2) overload around the IP joint with weight-bearing secondary to the immobile MTP joint, and (3) excessive pressure at the skin over the excrescence by shoe contact, which could be aggravated by the immobile MTP joint.

## 4. Conclusions

The correction of pronation deformity by hallux surgeries could enhance the medial bony excrescence at the medial aspect of the base of the distal phalanx of the hallux. This can cause symptomatic irritation between the excrescence and shoes. Since such occurrences are rare, we consider that there is no need to remove the excrescence in every case in the first surgical intervention.

## Figures and Tables

**Table 1:** Patients' backgrounds and radiographic measurements in the osteotomy and arthrodesis groups.

	Osteotomy (*n* = 97)	Arthrodesis (*n* = 33)
Sex F : M (% female)	89 : 8 (92%)	29 : 4 (88%)
Follow-up periods (months)	22.3 ± 13.4	20.3 ± 11.2
RA as underlying pathology (% patients)	30 (31%)	22 (67%)
Radiographic parameters		
Hallux valgus angle (degrees)		
Preoperative	40.5 ± 10.8	39.5 ± 22.5
Recent follow-up	11.5 ± 8.7^∗^	9.7 ± 7.0^∗^
Intermetatarsal angle (degrees)		
Preoperative	17.9 ± 4.3	15.0 ± 6.4
Recent follow-up	8.0 ± 4.3^∗^	11.2 ± 3.8^∗^
Width of excrescence (mm)		
Preoperative	0.9 ± 0.7	1.3 ± 0.8
Postop 1 month	1.5 ± 0.7^∗^	1.8 ± 1.0^∗^
Recent follow-up	1.4 ± 0.7^∗^	1.8 ± 0.7^∗^

RA: rheumatoid arthritis. All continuous variables were presented as mean ± standard deviation. A paired *t-*test was used to compare the radiographic measurements between preoperative and postoperative conditions. ^∗^*p* < 0.05 vs. preoperative condition.

## Data Availability

The data that support the findings of this study are available from the corresponding author [TM] upon reasonable request.
